# Synchronous action observation and motor imagery may not always represent the optimal form of action simulation: a commentary on Eaves et al. (2022)

**DOI:** 10.1007/s00426-023-01894-w

**Published:** 2023-11-08

**Authors:** David J. Wright, Paul S. Holmes

**Affiliations:** 1 Department of Psychology, Manchester Metropolitan University, Manchester, UK; 2 Department of Sport and Exercise Sciences, Manchester Metropolitan University, Manchester, UK

## Abstract

(Eaves et al., Psychological Research Psychologische Forschung, 2022) summary review, showing positive behavioural effects of AOMI interventions, is a welcome addition to the field. Several recent studies, however, have reported that AOMI may be no more beneficial than independent MI, and, for some tasks, may add no benefit beyond that obtained via physical practice. We discuss evidence to balance the narrative but support the pragmatic reasons why AOMI remains a suitable and appealing form of action simulation. We propose that further research interrogation of the discrete AOMI states through a more continuum-based approach could address some of the inconsistent data seen in AOMI research.

Eaves et al.’s (2022) review of combined action observation and motor imagery (AOMI) interventions is a welcome addition to the field. The authors present a summary of current literature investigating behavioural effects of AOMI interventions and conclude that AOMI is effective for facilitating behavioural outcomes, particularly during early stages of skill acquisition. We commend the authors for suggesting several novel research questions, including involving incorporation of established motor learning principles into AOMI research. These insightful proposals will help advance the field beyond examination of whether AOMI is effective, or more effective than motor imagery (MI) or action observation (AO), which has been investigated extensively.

Despite these positive contributions, the review could be argued to present a positively skewed account of the effectiveness of AOMI interventions. The authors are correct that AOMI can lead to beneficial behavioural effects, but several studies have shown AOMI to be no more effective than MI. For example, as noted by Eaves et al., Taube et al. (2014) conducted a four-week balance training study involving AOMI and MI intervention groups. Both interventions delivered improvements in balance, but there was no statistical difference between the groups, despite a trend for greater improvements via AOMI. Two additional studies, not acknowledged by Eaves et al., have reported similar effects. For example, Smith et al. (2020) conducted a single-case design study exploring bicep strength gains in untrained individuals, in which participants followed counterbalanced MI and AOMI interventions. The results showed beneficial effects from both interventions, but inclusion of video stimuli in the AOMI condition brought no benefits beyond those obtained via MI. Similarly, Marshall and Wright (2016) compared the effects of an AOMI intervention to a MI intervention developed via layered-stimulus response training on golf putting accuracy. Significant improvements were obtained in the MI group, but not the AOMI group. This appears to be the only AOMI study published to date to not show beneficial intervention effects. Taken together, these findings may indicate that the improved behavioural outcomes often reported in AOMI studies may be driven primarily by MI effects, with the AO component serving predominantly as a visual guide to support MI processes (Meers et al., 2020).

These findings from individual studies are reflected in a meta-analysis of behavioural effects of AOMI interventions, published by Chye et al. (2022) at the same time as Eaves et al.’s (2022) review. Chye et al. reported that AOMI interventions had a positive medium-to-large effect compared to control conditions, and a positive small-to-medium effect compared to AO interventions. AOMI interventions, however, were found to have no significant effect on movement outcomes when compared to MI. These findings do not necessarily challenge Eaves et al.’s support for AOMI, but rather serve to balance the argument and counter the narrative that AOMI always represents the optimal form of action simulation (see McNeill et al., 2020). Taken together, current evidence indicates that AOMI is effective for improving behavioural outcomes, but it appears to produce comparable, rather than superior, effects to MI (Chye et al., 2022; but see Romano-Smith et al., 2018). Despite this, there are pragmatic reasons why AOMI represents an appealing option for practitioners supporting skill acquisition. For example, the inclusion of video-based AO within AOMI allows the practitioner to ensure that the simulated visual content is demonstrated by an appropriately skilled model with the desired technique visual cues. It also offers control of the visual perspective, viewing angle, task relevant audio, and movement timing in a manner not possible directly through independent MI interventions (Holmes & Calmels, 2008; Wright et al., 2022). A more accurate conclusion, therefore, is that AOMI is at least equally effective as MI but provides numerous practical benefits which make it a more appealing choice in many situations.

There is also evidence, published since Eaves et al.’s review, indicating that, for certain tasks, AOMI may add no benefit beyond that obtained via physical practice. Scott et al. (2023) recruited children with developmental coordination disorder (DCD) for a four-week intervention involving the use of AOMI alongside physical practice to learn four activities of daily living. The AOMI group showed significant improvements in the learning of shoelace tying and cup stacking, compared to the control group who completed an equivalent amount of physical practice. The AOMI intervention was also particularly effective for learning shoelace tying in a sub-group of participants who were initially unable to complete this task at pre-test, indicating motor learning benefits. For the other skills assessed, cutlery use and shirt buttoning, the AOMI intervention delivered no measurable benefits beyond those obtained by the control group. As shoelace tying and cup stacking involve a greater number of different sub-components than the more repetitive actions of cutlery use or shirt buttoning, the findings were interpreted as evidence that AOMI alongside physical practice may only produce benefits over physical practice alone for the acquisition of more complex motor skills, at least for this DCD population. Further research exploring whether task-specific AOMI effects exist across different motor skill classifications and populations would represent an important addition to the literature.

A welcome aspect of Eaves et al.’s (2022) review is the inclusion of a glossary of key terms related to AOMI. Whilst this glossary should serve to improve consistency in terminology when reporting and describing AOMI studies, it may be an oversimplification to view congruent AOMI as when “the same action is observed and imagined simultaneously” (Eaves et al., 2022; p.3). Frank et al. (2020) argued that AOMI can never be truly congruent given that AOMI instructions typically require individuals to watch the video (visual modality) and imagine the feeling of the observed movement execution (kinaesthetic modality). Even accepting this paradox, a more nuanced view of the factors that influence the relative congruence of AOMI interventions is probably more appropriate. Within Eaves et al.’s definition for congruent AOMI, the level of congruence can vary depending on the content of the AO component and the wording of the MI instructions. For example, instructing kinaesthetic imagery alongside AO of a self-modelled action recorded from a first-person visual perspective is clearly more congruent than when the observed action is modelled by another person of a higher skill level, with different physical characteristics, and presented from a third-person visual perspective, yet both would be categorised as ‘congruent’ within the current framework. Rather than classifying these different scenarios as the same form of AOMI, it is more appropriate to consider a continuum of AOMI states, as originally proposed by Vogt et al. (2013) and more recently by Scott et al. (2022), in which observed and imagined actions can vary in their level of congruence based on many factors including: visual perspective and context, viewing presentation angle, movement timing, model similarity, model skill level, inclusion/exclusion of audio, and imagery ability characteristics. Research comparing the effects of AOMI interventions of varying levels of congruency between observed and imagined components may help establish a better understanding of the parameters under which AOMI represents an appropriate form of action simulation and may account for some of the conflicting findings described earlier.

In conclusion, Eaves et al. (2022) provide a positive summary of AOMI research and propose welcome suggestions for future research. We support the continued use of AOMI as one of the action simulation interventions available to researchers and practitioners but suggest that individual factors (e.g., an individual’s intervention modality preferences, imagery ability markers, and his/her level of expertise) and task factors (e.g., the task complexity and skill classification, and relative importance of technique or outcome for the specific skill) should determine the most appropriate simulation technique. Future AOMI research should investigate different motor skill classifications and different levels of congruency between observed and imagined components, along a continuum, to establish how this may influence effectiveness of AOMI interventions.

## Data Availability

Not applicable.
